# Gout and Risk of Ischemic Stroke in Patients With Atrial Fibrillation: A Nationwide Cohort Study

**DOI:** 10.1161/STROKEAHA.126.055194

**Published:** 2026-06-10

**Authors:** Antti Palomäki, Ville Langén, K.E. Juhani Airaksinen, Olli Halminen, Jari Haukka, Jussi Jaakkola, Elis Kouki, Birgitta Salmela, Jukka Putaala, Miika Linna, Pirjo Mustonen, Aapo L. Aro, Juha Hartikainen, Mika Lehto, Konsta Teppo

**Affiliations:** 1 Department of Rheumatology (A.P.), Turku University Hospital and University of Turku, Finland.; 2 Division of Medicine (A.P., V.L.), Turku University Hospital and University of Turku, Finland.; 3 Department of Geriatric Medicine (V.L.), Turku University Hospital and University of Turku, Finland.; 4 Turku University Hospital and University of Turku, Finland (K.E.J.A., P.M.).; 5 University of Eastern Finland, Kuopio (O.H.).; 6 University of Helsinki, Finland (J. Haukka, E.K., J.P., A.L.A., M. Lehto).; 7 Cardiac Unit, Department of Internal Medicine, Satasairaala, Pori, Finland (J.J.).; 8 Heart Center, Department of Internal Medicine, Päijät-Häme Central Hospital, Lahti, Finland (B.S.).; 9 Department of Neurology, Helsinki University Hospital, Finland (J.P.).; 10 University of Eastern Finland, Kuopio (M. Linna).; 11 Aalto University, Espoo, Finland (M. Linna).; 12 Heart and Lung Center, Helsinki University Hospital (A.L.A.).; 13 Kuopio University Hospital (J. Hartikainen).; 14 University of Eastern Finland (J. Hartikainen).; 15 Jorvi Hospital, Department of Internal Medicine, HUS Helsinki University Hospital (M. Lehto).; 16 Cardiac Unit, Department of Internal Medicine, Satasairaala, Pori, Finland (K.T.).; 17 Department of Internal Medicine, University of Turku, Finland (K.T.).

**Keywords:** anticoagulant, atrial fibrillation, gout, ischemic stroke, risk factor

## Abstract

**BACKGROUND:**

Gout is an emerging cardiovascular risk factor. We aimed to assess whether gout is associated with an increased risk of stroke in patients with atrial fibrillation (AF).

**METHODS:**

The nationwide registry-linkage FinACAF study (Finnish Anticoagulation in Atrial Fibrillation) included all patients with AF in Finland between 2007 and 2018 from all levels of care. Based on diagnosis codes and pharmacy claims data, the association of gout and urate-lowering therapy with the incidence of ischemic stroke was assessed.

**RESULTS:**

We identified 229 565 patients with new-onset AF (50.0% female; mean age, 72.7 years; mean follow-up, 4.0 years), of whom 6 910 (3.0%) had a history of gout. A total of 16 296 (7.1%) patients experienced an ischemic stroke. Gout was associated with higher stroke rates in both unadjusted and adjusted analyses (incidence rate ratio, 1.35 [95% CI, 1.22–1.49] and incidence rate ratio, 1.12 [95% CI, 1.02–1.24], respectively). Analyses restricted to follow-up without anticoagulation yielded consistent results with slightly higher point estimates (incidence rate ratio, 1.88 [95% CI, 1.63–2.17] unadjusted; incidence rate ratio 1.26 [95% CI, 1.09–1.46] adjusted). In patients with gout and AF, time-dependent exposure to urate-lowering therapy was associated with a 30% lower stroke rate. Nonanticoagulated crude stroke rates were 1.5, 1.0, and 4.8 per 100 patient-years for gout patients with CHA_2_DS_2_-VA scores of 0, 1, and ≥2, respectively.

**CONCLUSIONS:**

Gout is an important risk factor for ischemic stroke in patients with AF, and considering gout could improve stroke risk stratification. Urate-lowering therapy was associated with reduced stroke risk, suggesting that gout is a modifiable risk factor in patients with AF.

**REGISTRATION:**

URL: https://www.clinicaltrials.gov; Unique identifier: NCT04645537.

Atrial fibrillation (AF) is the most common cardiac arrhythmia, affecting up to 5.2% of the adult population.^1^ It is a major cause of ischemic stroke, with the risk of stroke varying considerably among individuals based on their specific comorbidities and other characteristics.^2,3^ With optimal therapy, including oral anticoagulant (OAC) treatment as well as management of relevant comorbidities, the risk of stroke can be significantly reduced.^4^ Identifying patients who would benefit from OAC therapy and have modifiable stroke risk factors is therefore essential for improving their prognosis.

Gout is the most common form of inflammatory arthritis with increasing prevalence globally.^5^ It is characterized by recurrent painful flares caused by an inflammatory reaction against monosodium urate crystals deposited in joints and surrounding tissues as a result of hyperuricemia.^6^ Gout has been associated with the risk of cardiovascular events, including myocardial infarction, stroke, and venous thromboembolism.^7–10^ AF is common in patients with gout and hyperuricemia, and patients with gout often share many risk factors for AF, particularly older age, male sex, obesity, hypertension, chronic kidney disease, and alcohol use.^11–14^ Gout is also often undertreated, with both low rates of urate-lowering therapy initiation and poor treatment adherence.^15^

In patients with AF, it remains uncertain whether gout contributes to an additional and potentially modifiable risk of stroke. Moreover, while there is some evidence that long-term urate-lowering therapy may lower the risk of acute coronary syndrome and stroke in patients with gout, whether this also applies to stroke risk in patients with coexisting AF and gout is unknown.^16–18^ These questions are clinically important, as appropriate management of gout might offer a relatively simple and cost-effective strategy to improve outcomes in patients with AF. Therefore, we conducted a nationwide retrospective cohort study to examine the association of gout with ischemic stroke in patients with AF. Additionally, we explored whether urate-lowering therapy is associated with a reduced stroke risk in patients with gout and AF.

## Methods

### Data Availability Statement

Because of the sensitive nature of the data collected for this study, requests to access the data set from qualified researchers trained in human subject confidentiality protocols may be sent to the Finnish national register holders (Social Insurance Institution of Finland, Finnish Institute for Health and Welfare, Population Register Center, and Tax Register) through Findata (https://findata.fi/en/). In the interest of research transparency and reproducibility, the analysis code used in this study has been made publicly available on GitHub and permanently archived on Zenodo under DOI 10.5281/zenodo.17228485. It can be accessed directly online at https://doi.org/10.5281/zenodo.17228485.

### Study Population

The FinACAF study (Finnish Anticoagulation in Atrial Fibrillation; ENCePP Identifier: EUPAS29845) is a nationwide retrospective cohort study that includes all patients documented with AF in Finland from 2004 to 2018.^19^ Patients were identified using all available national healthcare registers, including hospitalizations and outpatient specialist visits, and primary healthcare. and the National Reimbursement Register maintained by the Social Insurance Institute. The cohort inclusion criterion was an *International Classification of Diseases, Tenth Revision* diagnosis code of I48, encompassing AF and atrial flutter, collectively referred to as AF, recorded between 2004 and 2018. Exclusion criteria encompassed permanent emigration abroad before December 31, 2018, and age below 20 years at AF diagnosis. The present substudy was conducted within a cohort of patients with incident AF from 2007 to 2018, established in previous studies of the FinACAF cohort.^20–22^ The patient selection process is summarized in Figure S1.

### Follow-Up

The follow-up period was evaluated using 2 distinct approaches. In both strategies, the follow-up started from the first diagnosis of AF. In the main approach, follow-up continued until the occurrence of the first ischemic stroke event, death, or the end of the observation period on December 31, 2018, whichever occurred first. In this approach, the regressions were adjusted for the use of OACs in a time-dependent manner. Moreover, since it is the nonanticoagulated stroke rate that drives the clinical decision-making regarding stroke prevention with OACs, the second approach focused exclusively on the follow-up without OAC therapy.^23^ Thus, in the second approach, the follow-up ended on the first OAC purchase, the first stroke event, death, or the end of the observation period, whichever occurred first.

### Definition of Gout

Patients were classified as having gout if they had recorded gout diagnosis codes (*International Classification of Diseases, Tenth Revision*: M10 or International Classification of Primary Care, Second Edition: T92) in any of the nationwide hospital or primary care registers before or at the date of the first AF diagnosis. Furthermore, to explore gout severity and gauge potential causality between gout and stroke (on the assumption that a causal risk factor should show stronger associations with greater severity), we classified patients into 2 groups: those with a hospital-recorded diagnosis of gout (a surrogate for more severe disease requiring hospital-level care) and those with gout diagnosis recorded only in primary care (a surrogate for less severe disease). Additionally, gout patients were categorized into those with a pharmacy purchase of urate-lowering therapy (allopurinol or febuxostat) within the year before their first AF diagnosis and those without urate-lowering drug purchases.

### Exposure to Urate-Lowering Therapy

We considered allopurinol and febuxostat, the most commonly used urate-lowering therapies in Finland, in our analyses. First, we assessed stroke risk in patients with gout with and without these drug purchases at baseline, defined as at least 2 pharmacy purchases of allopurinol or febuxostat within the year before the first AF diagnosis (start of follow-up), including the date of diagnosis. Second, among patients with gout, we analyzed the effect of these drugs on stroke risk using a time-dependent exposure definition. In this approach, exposure to urate-lowering therapy began at the first pharmacy purchase occurring within 1 year before or any time after cohort entry and was assumed to continue until 120 days after the last recorded purchase. Follow-up with exposure to urate-lowering therapy was then compared with time without urate-lowering therapy. Purchases made >1 year before cohort entry were not considered as therapy initiation. The 120-day interval was chosen because, in Finland, medications can be reimbursed for up to 90 days at a time, with an additional 30-day grace period allowed to account for potential stockpiling and waning of the urate-lowering effect.

### Definition of Ischemic Stroke

In patients without prior ischemic stroke before the first AF diagnosis, an ischemic event was considered to occur on the first date of a recorded I63 or I64 *International Classification of Diseases, Tenth Revision* diagnosis code in the hospital care register after the cohort entry. In patients with prior ischemic stroke, the event was considered to occur on the date of the first new hospitalization with I63 or I64 *International Classification of Diseases, Tenth Revision* code as the main diagnosis, with at least a 90-day gap from the prior event, which had occurred before AF diagnosis.

### Study Ethics

The study protocol was approved by the Ethics Committee of the Medical Faculty of Helsinki University, Helsinki, Finland (nr. 15/2017 and 15/2024), and received research permission from the Helsinki University Hospital (HUS/46/2018 and HUS/217/2024). Respective permissions were obtained from the Finnish register holders (KELA 138/522/2018; Finnish Institute for Health and Welfare 2101/5.05.00/2018; Population Register center VRK/1291/2019-3; Statistics Finland TK-53-1713-18/ u1281; and Tax Register VH/874/07.01.03/2019). Patients’ personal identification numbers were pseudonymized, and the research group received individualized but unidentifiable data. Informed consent was waived due to the retrospective registry nature of the study. The study conforms to the Declaration of Helsinki as revised in 2024. This study is reported in accordance with the STROBE guidelines (Strengthening the Reporting of Observational Studies in Epidemiology; Supplemental Material).

### Statistical Analyses

We calculated incidence rates and incidence rate ratios (IRRs) for ischemic stroke using the Poisson regression model. The model employed a Lexis-type data structure, incorporating 2 time scales: follow-up time from AF diagnosis and age.^24^ This statistical approach was selected to address age progression over the relatively long observation period (2007–2018). Adjusted IRRs accounted for age (categorical variable with 10-year intervals), calendar year period, sex, heart failure, diabetes, hypertension, prior ischemic stroke, vascular disease, dyslipidemia, prior bleeding, alcohol use disorder, renal failure, cancer, dementia, psychiatric disorders, and income level (divided into tertiles). The definitions of the comorbidities are presented in Table S1. In analyses that also included follow-up with anticoagulation, OAC use was treated in a time-dependent manner, with treatment initiation marked by the first OAC purchase and continuation until 120 days after the last drug purchase. Additionally, we assessed the association between urate-lowering therapy (allopurinol or febuxostat) exposure and stroke risk among patients with gout. In these analyses, urate-lowering therapy use was modeled in a time-dependent manner (as detailed above in the exposure to urate-lowering therapy paragraph), and adjusted models included the aforementioned variables, including OAC use. Sensitivity analyses were conducted among patients without baseline stroke, as first-ever strokes may be more reliably defined than recurrent events in administrative registry data. Moreover, we assessed whether the association between gout and stroke risk differed across stroke risk categories by fitting a Poisson regression model including gout, stroke risk category (3 groups: CHA_2_DS_2_-VA scores 0, 1, or ≥2), and their interaction term. Baseline variables were compared using the χ^2^ test, Student *t* test, and ANOVA. Standardized mean differences of baseline variables are also reported. All tests were 2-sided, with statistical significance assessed using a *P* value threshold of 0.05 or the 95% CIs. Statistical analyses were conducted using IBM SPSS Statistics software version 28.0 (SPSS Inc, Chicago, IL) and R version 4.0.5 (R Core Team, Vienna, Austria; https://www.R-project.org).

## Results

We identified 229 565 patients with new-onset AF (50.0% female; mean age, 72.7 years; mean follow-up time, 4.0 years). Overall, 6 910 patients (3.0%) had a history of gout, of whom 3 796 (1.7%) were diagnosed at the hospital level, and 3 114 (1.4%) had a gout diagnosis recorded only in primary care. Of the patients with gout, 2 978 (43.1%) had purchased urate-lowering therapies within a year before the first AF diagnosis. Patients with gout had a higher overall prevalence of comorbidities than patients without gout, which was also reflected in their higher stroke and bleeding risk scores (Table). Of all patients with gout, 103 (1.5%), 443 (6.4%), and 6 364 (92.1%) were classified as low (CHA_2_DS_2_-VA=0), moderate (CHA_2_DS_2_-VA=1), and high (CHA_2_DS_2_-VA≥2) stroke risk, respectively. Patients with gout diagnosed in a hospital setting had a higher prevalence of comorbidities compared with those diagnosed only in primary care. Similarly, patients with urate-lowering medication at baseline had more comorbidities than those without urate-lowering treatment (Table S2). None of the patients in the cohort used colchicine at baseline. Patients with gout were more likely to initiate OAC therapy during the follow-up period, compared with patients without gout (73.7% versus 70.4%; *P*<0.001). Moreover, mortality during follow-up was higher in those with gout than in those without gout (35.0% versus 33.2%; *P*<0.001).

**Table.**
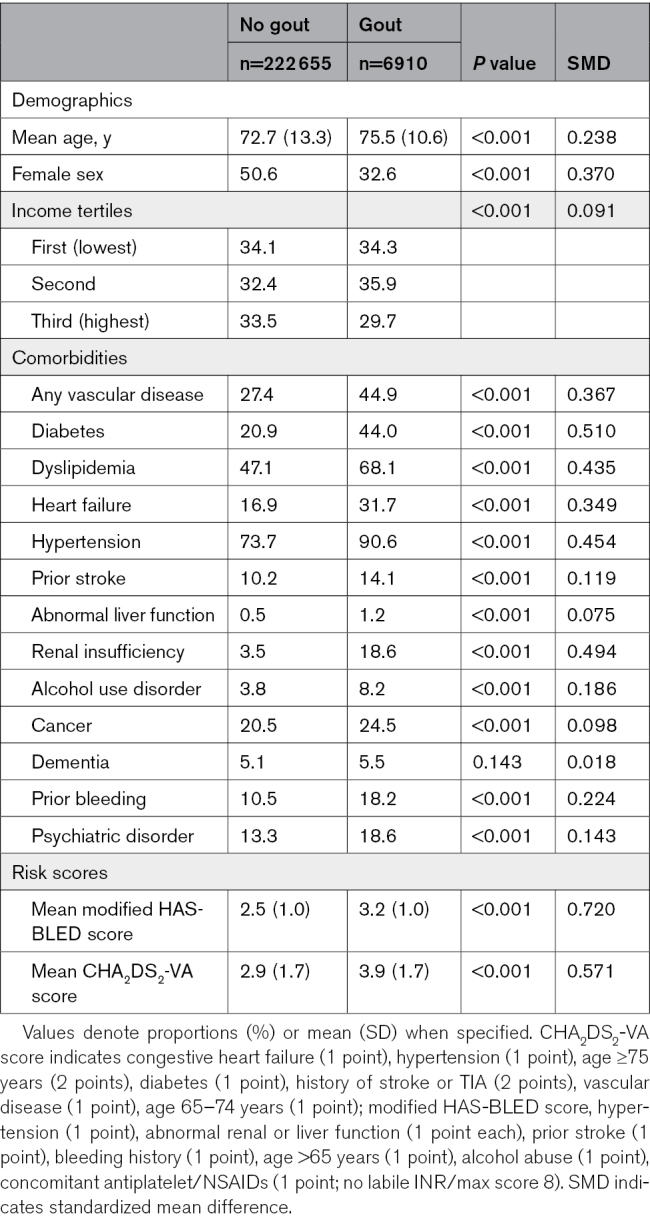
Baseline Characteristics of the Study Cohort According to the History of Gout

**Figure 1.**
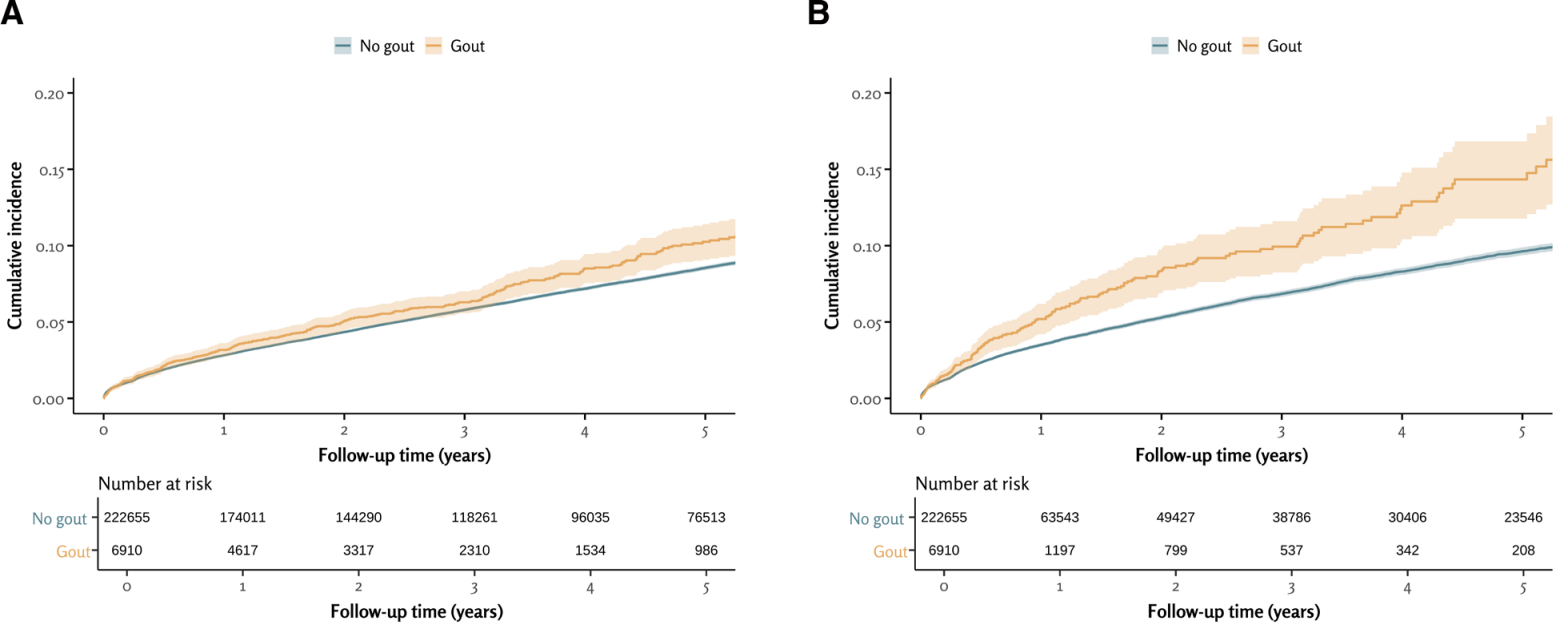
**Cumulative incidence of ischemic stroke in patients with atrial fibrillation.** Cumulative incidence of ischemic stroke in patients with atrial fibrillation according to the presence of gout for the entire follow-up (**A**) and for the follow-up without anticoagulation (**B**). Shaded areas represent 95% CIs.

**Figure 2.**
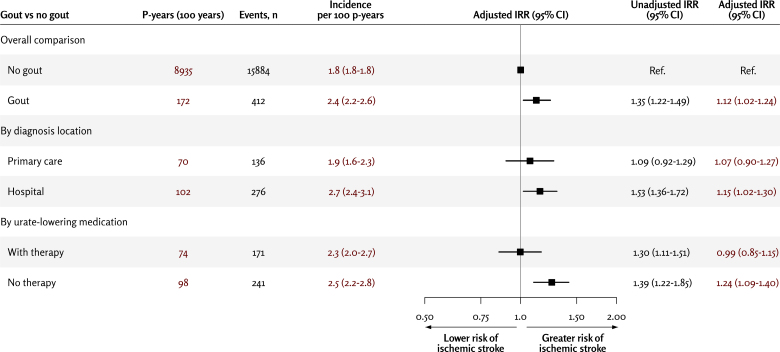
**Ischemic stroke rates in patients with and without gout.** Incidence rate ratios (IRRs) estimated with Poisson regression and adjusted for age, sex, calendar year, heart failure, diabetes, hypertension, prior ischemic stroke, vascular disease, dyslipidemia, prior bleeding, alcohol use disorder, renal failure, cancer, dementia, psychiatric disorders, income level, and anticoagulant use. P-year indicates patient-year.

A total of 16 296 (7.1%) patients suffered an ischemic stroke during the entire follow-up period. Gout was associated with an elevated stroke rate both in the unadjusted and adjusted analyses (IRR, 1.35 [95% CI, 1.22–1.49] and IRR, 1.12 [95% CI, 1.02–1.24], respectively; Figures 1 and 2). Similarly, gout was associated with stroke risk in the unadjusted and adjusted sensitivity analyses restricted to patients without prior stroke (IRR, 1.36 [95% CI, 1.22–1.51] and IRR, 1.14 [95% CI,1.02–1.27], respectively). The association with ischemic stroke was stronger in patients with a hospital-level diagnosis of gout, whereas the association was smaller and statistically nonsignificant in those with only a primary care diagnosis of gout. No interaction between gout and stroke risk category was observed for stroke rate, suggesting a consistent relative association between gout and stroke risk across all categories (interaction *P*=0.570).

When only follow-up without anticoagulation was analyzed, the findings were consistent with those of the analysis including follow-up with OAC use, although the risk point estimates were higher: gout was associated with a higher ischemic stroke risk in both unadjusted and adjusted analyses (IRR, 1.88 [95% CI, 1.63–2.17] and IRR, 1.26 [95% CI, 1.09–1.46], respectively). When patients were categorized based on their CHA_2_DS_2_-VA score, the crude nonanticoagulated stroke rates were 1.5, 1.0, and 4.8 strokes per 100 patient-years for patients with a CHA_2_DS_2_-VA score of 0, 1, and 2 or more, respectively (Figure S2).

**Figure 3.**
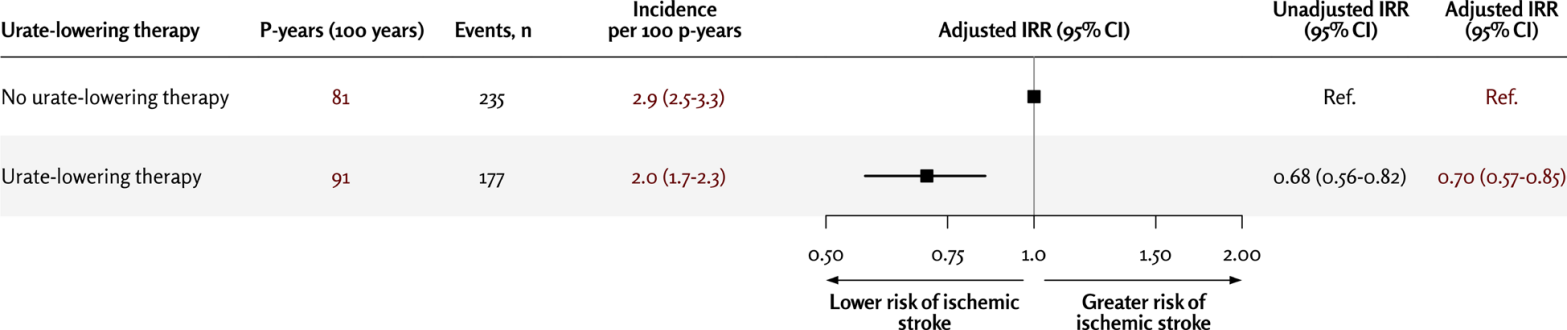
**Ischemic stroke rates in patients with gout and atrial fibrillation according to time-dependent urate-lowering medication exposure.** Incidence rate ratios (IRRs) estimated with Poisson regression and adjusted for age, sex, calendar year, heart failure, diabetes, hypertension, prior ischemic stroke, vascular disease, dyslipidemia, prior bleeding, alcohol use disorder, renal failure, cancer, dementia, psychiatric disorders, income level, and anticoagulant use. Urate-lowering medication is considered time-dependent. P-year indicates patient-year.

When patients with gout were categorized by urate-lowering therapy use at baseline, those with and without urate-lowering therapy both had higher crude stroke rates compared with patients without gout. However, in adjusted analyses, the association between gout and ischemic stroke was evident only among those without urate-lowering therapy, whereas no association was observed among those with urate-lowering therapy compared with patients without gout (Figure 2). When patients with gout who were not receiving urate-lowering therapy were used as reference, urate-lowering therapy at baseline was associated with a significantly lower stroke rate (adjusted IRR, 0.80 [95% CI, 0.66–0.98]). In sensitivity analyses among patients without prior stroke, a similar, though statistically nonsignificant, trend toward lower stroke rates with baseline urate-lowering therapy was observed (adjusted IRR, 0.84 [95% CI, 0.68–1.04]). Among patients with both gout and AF, when exposure to urate-lowering therapy was considered time-dependently, it was associated with a lower stroke rate in both unadjusted and adjusted analyses (Figure 3). This finding was consistent when analyses were restricted to patients without prior stroke (unadjusted and adjusted IRRs, 0.66 [95% CI, 0.54–0.82] and 0.66 [95% CI, 0.53–0.82], respectively).

## Discussion

This nationwide retrospective cohort study demonstrated that gout is associated with a 12% to 26% higher risk of ischemic stroke in patients with AF. Patients with gout who were categorized as having low or moderate stroke risk based on their CHA_2_DS_2_-VA score exhibited an annual nonanticoagulated stroke rate of ≥1%. Moreover, urate-lowering therapy was associated with an ≈30% lower stroke risk in patients with gout, and those receiving urate-lowering therapy had a stroke risk comparable to patients without gout.

Previous data on the association between gout and ischemic stroke risk in patients with AF are scarce. One cross-sectional study based solely on hospital-level data from US registries reported a 10% higher odds of stroke associated with gout.^25^ However, the generalizability of its findings is limited by the lack of adjustment for OAC therapy and the potential for substantial selection bias. Nevertheless, the findings of the present study align with this earlier observation. Of note, the 12% to 26% higher stroke risk associated with gout in the current study is clinically meaningful and comparable in magnitude to that observed for several established stroke risk factors in patients with AF, such as diabetes, hypertension, vascular disease, and heart failure.^21,22,26–28^ Moreover, our study provides some evidence supporting a potential causal dose–response relationship between gout and stroke, as patients with gout requiring hospital-level treatment had a higher stroke risk than those with gout recorded only in primary care registries. Our findings are also consistent with previous studies in patients without AF, showing that gout flares are associated with a significantly increased risk of cardiovascular events, including stroke.^7,8^ Since ischemic events in patients with AF are often cardioembolic, a previous finding that gout is associated with increased risk of venous thromboembolism might also be significant.^9^

Patients with gout who received urate-lowering therapy at baseline had a stroke risk comparable to patients without gout in the adjusted analyses, and notably lower than that of patients with gout who were not receiving urate-lowering therapy. Correspondingly, when exposure to urate-lowering therapy was considered in a time-dependent manner among patients with both gout and AF, treatment exposure was associated with an approximately one-third lower stroke rate (Figure 3). To the authors’ knowledge, no prior studies have examined the association between urate-lowering therapy and stroke risk specifically in patients with both gout and AF. However, our findings are concordant with some previous observational studies showing that long-term urate-lowering therapy is associated with a lower risk of stroke and acute coronary syndrome in patients with gout.^16,17,29,30^ A recent target trial emulation study also demonstrated that achieving guideline-recommended serum urate levels was associated with a reduced risk of cardiovascular events, including stroke, in patients with gout.^18^ Although current AF guidelines do not recognize gout as an established stroke risk factor, accumulating evidence, including findings from the present study, suggests that management of gout may contribute to improving cardiovascular outcomes. Notably, these potential benefits appear confined to patients with gout, as urate-lowering therapy has not demonstrated cardiovascular benefits in randomized trials among patients without gout.^31^ Hence, hyperuricemia alone may not fully explain the increased cardiovascular risk in gout or the elevated stroke risk observed in patients with AF in this study. Instead, the hyperinflammation and thrombogenic processes associated with gout and its flares may play a more central role.^9,32,33^ Inflammatory biomarkers in patients with gout have been shown to gradually decrease with urate-lowering therapy.^34^ The inflammation hypothesis is also supported by the positive results of colchicine prophylaxis for gout flares, which simultaneously decreases the risk of cardiovascular events.^35^ In summary, although residual confounding by indication bias cannot be excluded in observational studies, our results suggest that gout may represent a modifiable stroke risk factor in patients with AF.

Gout is strongly associated with metabolic and cardiovascular comorbidities, a pattern also evident in the characteristics of patients with gout in the present study.^6,11^ Relatedly, when applying the CHA_2_DS_2_-VA score, the vast majority of patients with AF and gout were classified as high stroke risk, while only a small fraction were categorized as low (1.5%) or moderate (6.4%) risk. Thus, for most patients with gout and AF, age and comorbidity burden already provide a clear indication for OAC therapy. However, an important finding was that patients with gout and CHA_2_DS_2_-VA scores of 0 to 1 also exhibited an elevated stroke risk, with annual nonanticoagulated rates of 1% or more, therefore exceeding the threshold above which OAC therapy is estimated to provide a net benefit in AF.^36–38^ It is worth noting, however, that these subgroups of gout patients with lower stroke risk scores were relatively small, and these findings should be interpreted cautiously as hypothesis-generating. Nevertheless, despite wide CIs in the lower-risk categories, the interaction analyses suggested a consistent relative association between gout and stroke risk across these risk categories. Thus, gout could be considered in stroke risk stratification in addition to the conventional scores, particularly for patients classified as low risk who might otherwise not be considered for OAC therapy.

A key strength of the present study is its nationwide coverage of all patients diagnosed with AF across all levels of care, providing a uniquely comprehensive perspective on gout and stroke, improving the generalizability of the findings.^19^ Additionally, the hospital care register used to define ischemic stroke events is well-validated and has high diagnostic accuracy, particularly regarding cardiovascular diseases.^39^ Nevertheless, the limitations of our study need to be acknowledged, the most important of which are the challenges inherent in register-based retrospective cohort studies. Thus, information bias may be present in the administrative data due to inaccurate recording. Likewise, although urate-lowering therapy exposure was based on comprehensive pharmacy claims data, it is unknown whether patients actually took the medications. Moreover, importantly, our results reflect associations, and not necessarily causal relationships between gout, urate-lowering treatment, and stroke. Additionally, data were unavailable on some relevant variables, including urate levels, estimated glomerular filtration rate, smoking status, number of prior gout attacks, detailed measures of gout severity, stroke cause, blood pressure, and serum lipid and glycemic parameters. In Finland, colchicine use for gout has been limited by cost, lack of reimbursement, and the absence of official marketing authorization, and no patients in our cohort used the drug, precluding analysis of its effects. Finally, although the linked registry data allowed adjustment for numerous potential confounders, residual confounding from unmeasured factors cannot be excluded, including dynamic changes in the presence and severity of gout, as well as the onset of other comorbidities and changes in drug therapy.

## Conclusions

This nationwide cohort study showed that gout was associated with a significantly higher risk of ischemic stroke in patients with AF. Considering gout in addition to conventional risk scores could improve stroke risk stratification. Finally, urate-lowering therapy was associated with a lower risk of stroke in patients with gout, which supports guideline-recommended treatment of hyperuricemia in these patients and suggests that gout may be a modifiable risk factor for stroke in patients with AF.

## ARTICLE INFORMATION

### Sources of Funding

### Disclosures

Dr Langén reports consultant fees from Boehringer Ingelheim and grants from the State Research Funding of the wellbeing services county of Southwest Finland. Dr Palomäki reports consultant fees from Boehringer Ingelheim and AbbVie; compensation for other services from AbbVie, Pfizer, Johnson & Johnson, UCB, Lilly, and Boehringer Ingelheim; and travel support from Novartis. Dr Putaala reports grants from Amgen, Bayer, the Finnish Foundation for Cardiovascular Research, and the Sigrid Juselius Foundation; consultant fees from AstraZeneca and PeerVoice; and stock ownership in Vital Signum. Dr Mustonen reports consultant fees from Roche Health Solutions Inc and travel support from Pfizer. Dr Airaksinen reports consultant fees from Boehringer Ingelheim and Bayer, and grants from the Clinical Research Fund of Turku University Hospital and the Finnish Foundation for Cardiovascular Research. Dr Lehto reports consultant fees from Bayer, BMS, Pfizer, MSD, and Boehringer Ingelheim; and grants from the Finnish Foundation for Cardiovascular Research, the Aarne Koskelo Foundation, and the Helsinki and Uusimaa Hospital District research fund. Dr Salmela reports travel support from Pfizer. Dr Aro reports grants from Sydäntutkimussäätiö. The other authors report no conflicts.

### Supplemental Material

Tables S1–S2

Figures S1–S2

STROBE Checklist

## Supplementary Material
