# Supplementary material for: Gout and Risk of Ischemic Stroke in Patients With Atrial Fibrillation: A Nationwide Cohort Study
Source: Stroke. 2026 Jun 10;57(8):2400–7. doi: 10.1161/STROKEAHA.126.055194 (PMC13399721; doi:10.1161/STROKEAHA.126.055194)
Supplement: Supplementary file 1 [file str-57-2400-s001.pdf]

Supplemental Material

Table S1. Definitions of the comorbidities

Table S2. Baseline characteristics of the study cohort according to the diagnosis level of gout and use of urate-lowering therapy at baseline

Figure S1. Flow-chart of the patient selection process

Figure S2. Ischemic stroke rates according to the CHA_2DS_2-VA score during the entire follow-up and during the follow-up without anticoagulation

Table S1. Definitions of the comorbidities

	ICD-10	ICPC-2	Reimbursement code	ATC code	Other
Any vascular disease	I20-I25, I65-I66, I67.2, I70	K74, K75, K76, K91, K92	206		
Cancer					Any cancer registered in the Finnish Cancer Registry
Diabetes	E10-E14	T89, T90	103, 215	A10	
Dyslipidemia	E78	T93	206	C10	
Heart failure	I50, I11.0, I13.0, I13.2	K77	201		
Hypertension	I10-I15	K85 K86 K87	205	C03A, C03B, C03DB, C03EA,C07A, C08CA, C08D, C09	
Previous stroke	I63, I64, I69.3-I69.8	K90			
Bleeding history	D50.0, D62, D68.3, I60-I62, I69.0-I69.2, I85.0, I86.4, J94.2, K22.1, K22.3, K22.6, K25.0, K25.2, K25.4, K25.6, K26.0, K26.2, K26.4, K26.6, K27.0, K27.2, K27.4, K27.6, K28.0, K28.2, K28.4, K28.6, K29.0, K62.5, K63.1, K63.3, K92.0-K92.2, N02, R04, R31, R58, S06.2-S06.6, S06.8				
Alcohol use disorder	F10				
Renal failure	N18, Z49				
Liver cirrhosis or failure	K70.2-K70.4, K71.7, K71.8, K72, K74				
Dementia	F00-F03, G30				
Psychiatric disorder	F04-F99				

Abbreviations: ATC, anatomic therapeutic chemical; ICD-10, International Classification of Diseases, Tenth Revision; ICPC-2, International Classification of Primary Care, Second Edition

Table S2. Baseline characteristics of the study cohort according to the diagnosis level of gout and use of urate-lowering therapy at baseline

	No gout	Gout (hospital diagnosis)	Gout (primary care diagnosis only)	P-value	Gout (with urate-lowering therapy	Gout (without urate-lowering therapy)	P-value
	n=222 655	n=3 796	n=3 114		n=2 978	n=3 932	
**Demographics**							
Mean age, years	72.7 (13.3)	75.9 (10.9)	75.0 (10.2)	<0.001	76.2 (10.3)	75.0 (10.8)	<0.001
Female sex	50.6	33.4	31.7	<0.001	33.9	31.6	<0.001
**Income quartiles**				<0.001			<0.001
1st (lowest)	34.1	38.8	38.8		37.2	32.1	
2nd	32.4	34.9	34.9		35.2	36.5	
3rd (highest)	33.5	26.3	26.3		27.6	31.3	
**Comorbidities**							
Any vascular disease	27.4	47.8	41.3	<0.001	47.4	42.9	<0.001
Diabetes	20.9	44.9	43.0	<0.001	50.8	38.9	<0.001
Dyslipidemia	47.1	66.3	70.4	<0.001	71.0	65.9	<0.001
Heart failure	16.9	37.2	25.0	<0.001	38.3	26.7	<0.001
Hypertension	73.7	90.3	91.0	<0.001	93.1	88.7	<0.001
Prior IS	10.2	15.4	12.5	<0.001	13.8	14.3	<0.001
Abnormal liver function	0.5	1.6	0.6	<0.001	1.4	0.9	<0.001
Alcohol use disorder	3.8	9.1	7.1	<0.001	8.2	8.2	<0.001
Cancer	20.5	24.7	24.3	<0.001	25.7	23.7	<0.001
Dementia	5.1	6.8	4.0	<0.001	5.4	5.6	0.033
Prior bleeding	10.5	20.4	15.6	<0.001	18.8	17.8	<0.001
Psychiatric disorder	13.3	18.5	18.7	<0.001	18.3	18.8	<0.001
**Risk scores**							
Mean modified HAS-BLED score	2.5 (1.0)	3.2 (1.1)	3.1 (0.9)	<0.001	3.3 (1.0)	3.1 (1.0)	<0.001
Mean CHA_2DS_2-VA score	2.9 (1.7)	4.0 (1.7)	3.7 (1.6)	<0.001	4.1 (1.6)	3.7 (1.7)	<0.001

Values denote proportions (%) or mean (standard deviation). Abbreviations: CHA_2DS_2-VASc score, congestive heart failure (1 point), hypertension (1 point), age ≥75 years (2 points), diabetes (1 point), history of stroke or TIA (2 points), vascular disease (1 point), age 65-74 years (1 point); IS, ischemic stroke; modified HAS-BLED score, hypertension (1 point), abnormal renal or liver function (1 point each), prior stroke (1 point), bleeding history (1 point), age >65 years (1 point), alcohol abuse (1 point), concomitant antiplatelet/NSAIDs (1 point) (no labile INR, max score 8). p-values are for differences between the two gout categories and those without gout.

Figure S1. Flow-chart of the patient selection process

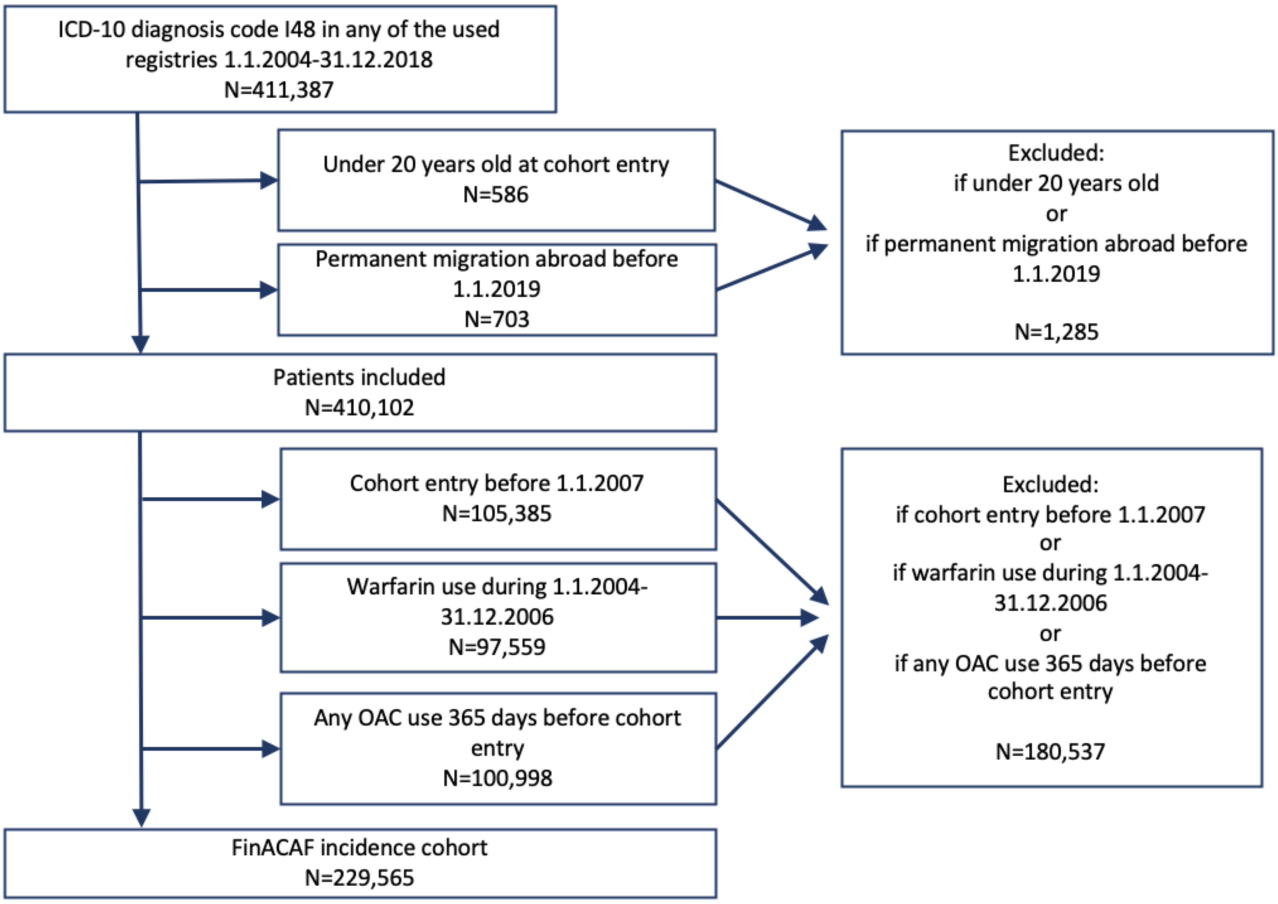

Figure S2. Ischemic stroke rates according to the CHA_2DS_2-VA score during the entire follow-up and during the follow-up without anticoagulation

Gout vs no gout	P-years (100 years)	Events, n	Incidence per 100 p-years	Unadjusted IRR (95% CI)	Adjusted IRR (95% CI)
Entire follow-up					
Low risk (CHA2DS2-VA = 0)					
No gout	1141	556	0.5 (0.5-0.5)	Ref.	Ref.
Gout	4	3	0.8 (0.2-2.4)	1.68 (0.54-5.22)	1.24 (0.40-3.88)
Moderate risk (CHA2DS2-VA = 1)					
No gout	1708	1396	0.8 (0.8-0.9)	Ref.	Ref.
Gout	16	18	1.1 (0.7-1.8)	1.38 (0.87-2.20)	1.25 (0.78-2.01)
High risk (CHA2DS2-VA ≥ 2)					
No gout	6085	13932	2.3 (2.3-2.3)	Ref.	Ref.
Gout	152	391	2.6 (2.3-2.8)	1.12 (1.01-1.24)	1.12 (1.01-1.24)
Only follow-up without anticoagulation					
Low risk (CHA2DS2-VA = 0)					
No gout	730	304	0.4 (0.4-0.5)	Ref.	Ref.
Gout	2	3	1.5 (0.3-4.5)	3.67 (1.18-11.43)	2.50 (0.80-7.82)
Moderate risk (CHA2DS2-VA = 1)					
No gout	775	689	0.9 (0.8-1.0)	Ref.	Ref.
Gout	6	6	1.0 (0.4-2.2)	1.14 (0.51-2.54)	0.92 (0.41-2.07)
High risk (CHA2DS2-VA ≥ 2)					
No gout	1593	5933	3.7 (3.6-3.8)	Ref.	Ref.
Gout	39	187	4.8 (4.2-5.6)	1.30 (1.12-1.50)	1.28 (1.10-1.48)

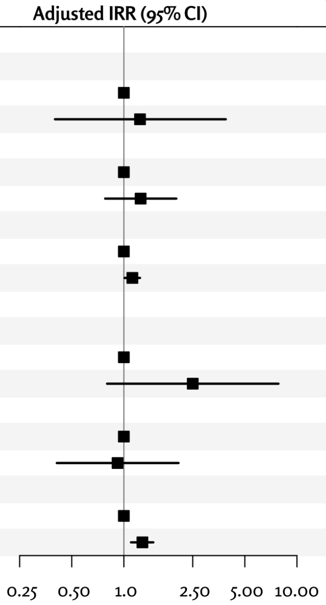

Footnote: Abbreviations: IRR, incidence rate ratio; P-year, patient-year. IRRs estimated with Poisson regression and adjusted for age, sex, calendar year, heart failure, diabetes, hypertension, prior ischemic stroke, vascular disease, dyslipidemia, prior bleeding, alcohol use disorder, liver cirrhosis or failure, cancer, dementia, psychiatric disorders, income level and use of anticoagulation
